# Informed interpretation of metagenomic data by StrainPhlAn enables strain retention analyses of the upper airway microbiome

**DOI:** 10.1128/msystems.00724-23

**Published:** 2023-11-02

**Authors:** Nadja Mostacci, Tsering Monika Wüthrich, Léa Siegwald, Silas Kieser, Ruth Steinberg, Olga Sakwinska, Philipp Latzin, Insa Korten, Markus Hilty

**Affiliations:** 1 Institute for Infectious Diseases, University of Bern, Bern, Switzerland; 2 Graduate School for Biomedical Science, University of Bern, Bern, Switzerland; 3 Nestlé Institute of Health Sciences, Nestlé Research, Société des Produits Nestlé S.A., Lausanne, Switzerland; 4 Division of Respiratory Medicine, Department of Pediatrics, Inselspital, University of Bern, Bern, Switzerland; Universita degli Studi di Trento, Trento, Italy

**Keywords:** respiratory tract, metagenomics, bacterial culture, strain resolution, genome analysis

## Abstract

Shotgun metagenomic sequencing has the potential to provide bacterial strain-level resolution which is of key importance to tackle a host of clinical questions. While bioinformatic tools that achieve strain-level resolution are available, thorough benchmarking is needed to validate their use for less investigated and low biomass microbiomes like those from the upper respiratory tract. We analyzed a previously published data set of longitudinally collected nasopharyngeal samples from Bangladeshi infants (Microbiota and Health study) and a novel data set of oropharyngeal samples from Swiss children with cystic fibrosis. Data from bacterial cultures were used for benchmarking the parameters of StrainPhlAn 3, a bioinformatic tool designed for strain-level resolution. In addition, StrainPhlAn 3 results were compared with metagenomic assemblies derived from StrainGE and newly derived whole-genome sequencing data. After optimizing the analytical parameters, we compared StrainPhlAn 3 results to culture gold standard methods and achieved sensitivity values of 87% (*Streptococcus pneumoniae*), 80% (*Moraxella catarrhalis*), 75% (*Haemophilus influenzae*), and 57% (*Staphylococcus aureus*) for 420 nasopharyngeal and 75% (*H. influenzae*) and 46% (*S. aureus*) for 260 oropharyngeal samples. Comparing the phylogenetic tree of the core genome of 50 *S*. *aureus* isolates with a corresponding marker gene tree generated by StrainPhlAn 3 revealed a striking similarity in tree topology for all but three samples indicating adequate strain resolution. In conclusion, a comparison of StrainPhlAn 3 results to data from bacterial cultures revealed that strain-level tracking of the respiratory microbiome is feasible despite the high content of host DNA when parameters are carefully optimized to fit low biomass microbiomes.

**IMPORTANCE:**

The usage of 16S rRNA gene sequencing has become the state-of-the-art method for the characterization of the microbiota in health and respiratory disease. The method is reliable for low biomass samples due to prior amplification of the 16S rRNA gene but has limitations as species and certainly strain identification is not possible. However, the usage of metagenomic tools for the analyses of microbiome data from low biomass samples is not straight forward, and careful optimization is needed. In this work, we show that by validating StrainPhlAn 3 results with the data from bacterial cultures, the strain-level tracking of the respiratory microbiome is feasible despite the high content of host DNA being present when parameters are carefully optimized to fit low biomass microbiomes. This work further proposes that strain retention analyses are feasible, at least for more abundant species. This will help to better understand the longitudinal dynamics of the upper respiratory microbiome during health and disease.

## INTRODUCTION

The development of high-throughput sequencing technologies has greatly facilitated the characterization of the human microbiome in health and disease. 16S rRNA gene amplicon sequencing has been used to characterize the human microbiota, including the one from the respiratory tract (1). Disordered microbiota patterns have been found in the respiratory tract of patients suffering from diseases with obvious involvement of microbes such as cystic fibrosis (CF) (2–4). Disordered microbiota patterns have also been observed in other conditions like asthma (5), chronic obstructive pulmonary disease (6–9), and interstitial lung disease (10), where bacterial respiratory infections are not known to be the main drivers of disease. However, 16S rRNA gene sequencing does not allow for the investigation of strain retention due to limited strain resolution within bacterial taxa.

Strain retention is usually investigated using traditional culturing methods combined with whole genome sequencing (WGS) from longitudinally collected isolates. As an example, convergent evolution and adaption of *Pseudomonas aeruginosa* and *Staphylococcus aureus* strains have been studied in patients with CF (11, 12). However, for most species, including mainly commensal or difficult to culture bacteria of the respiratory microbiome, strain retention remains understudied. Using shotgun metagenomic sequencing and appropriate bioinformatic tools could allow investigating strain retention of microbial species beyond selected culturable pathobionts. For example, StrainPhlAn has been developed to achieve microbial strain-level resolution using shotgun metagenomic data for more than 1,500 gut metagenomes (13). StrainPhlAn is based on reconstructing consensus sequence variants within species-specific marker genes and using them to estimate strain-level phylogenies. In contrast to other tools like StrainGE (14), short-read sequences do not have to be assembled into contigs. It is less clear if strain resolution can also be achieved on less investigated microbiomes, such as respiratory samples, which contain a high proportion of host-derived DNA (15). Therefore, using such strain-level resolution tools requires careful benchmarking to address low bacterial biomass (16).

Lower airway samples are considered to be clinically relevant for a number of respiratory diseases, but the sampling is invasive. However, it has been hypothesized that the lower airway microbiome is seeded by the oropharyngeal (OP) and nasopharyngeal (NP) microbiome (17).

Within this study, we aimed to optimize StrainPhlAn 3 to achieve strain resolution from low-biomass samples from the respiratory tract. To achieve this aim, we used culture and shotgun sequencing data from upper airway samples from a previously published NP data set from Bangladeshi infants (18) and a new data set from Swiss children with CF. To test the applicability of the approach on low biomass, we analyzed the retention over time of multiple strains of clinically relevant microbial species.

## RESULTS

### Data characteristics and bacterial species assignment

An NP and OP data set comprising both bacterial culture and shotgun metagenomic data were investigated for this study (Table 1). The read numbers of the NP data ranged from 40.1M reads to 186.5M reads, with a mean of 76.1M reads. After removing the human reads, the range of bacterial reads was between 0.3M and 74.8M, with a mean value of 11.7M. For the OP data, the number of reads varied between 14M and 199M with an average of 65.6M and between 0.07M and 158.7M with an average of 19.0M after removing human reads.

**TABLE 1:** Characteristics of data sets^*a*^

Type of samples	Age	*N* (individuals)	*N* (samples)	Sequencing information	Culture results	Disease	Origin	Reference
NP	2 mo	223	223	2 × 150 bp paired-end sequencing; Illumina HiSeq; 11.4 Gb (mean)	119 of 221 (54%; SP), 69 of 222 (31%; HI), 75 of 222 (33%; MC) 92 of 222 (41; SA)	H	BD	(18)
NP	4 mo	199	199	2 × 150 bp paired-end sequencing; Illumina HiSeq; 11.4 Gb (mean)	129 of 198 (65%; SP), 93 of 198 (47%; HI), 75 of 198 (38%; MC) 30 of 198 (15%; SA)	H	BD	(18)
OP	<4 yr	15	96	NovaSeq 6000 PE150; 10 G of raw data per sample	26 (27%; HI), 38 (40%; SA), 5 (5%; PA)	CF	CH	This study
OP	4–6 yr	15	87	NovaSeq 6000 PE150; 10 G of raw data per sample	31 (36%; HI), 36 (41%; SA), 5 (6 %; PA)	CF	CH	This study
OP	6–8 yr	14	60	NovaSeq 6000 PE150; 10 G of raw data per sample	19 (32%; HI), 34 (57%; SA), 1 (2%; PA)	CF	CH	This study
OP	>8 yr	8	16	NovaSeq 6000 PE150; 10 G of raw data per sample	8 (50%; HI), 15 (94%; SA)	CF	CH	This study

^*a*^ The total number of samples with available cultures from the NP samples are: *M. catarrhalis* (MC) (*N* = 378), *H. influenzae* (HI) (*N* = 380), *S. pneumoniae* (SP) (*N* = 377), and *S. aureus* (SA) (*N* = 375). *Pseudomonas aeruginosa* (PA) was only cultured for OP samples. For 1 OP sample, age was not available. Healthy individuals (H) and individuals with cystic fibrosis (CF) were included from Bangladesh (BD) and Switzerland (CH), respectively. Individuals were aged from 2 mo up to 10 yr.

Using the remaining bacterial reads of both data sets and comparing them with culture data, we first assessed species assignment (Table 2; Fig. S1). Compared to bacterial culture data of four species, MetaPhlAn 3 and Metagenome-Atlas showed comparable sensitivity, specificity, and F1 values (Table 2).

**TABLE 2:** Performance of bioinformatics tools at the species level^*a*^

Species (data set)	Analysis tool	TP^*b*^	TN	Specificity	Sensitivity	F1 score
*S. pneumoniae* (NP)	MetaPhlAn 3	240	121	71%	97%	0.89
	Metagenome-Atlas	224	102	66%	95%	0.88
	*StrainGE*	73	151	97%	31%	0.47
	*StrainPhlan 3 df*	168	154	90%	68%	0.78
	*StrainPhlan 3 new*	215	126	74%	87%	0.85
*S. aureus* (NP)	MetaPhlAn 3.0	87	257	86%	71%	0.70
	Metagenome-Atlas	74	253	90%	67%	0.70
	*StrainGE*	10	275	98%	9%	0.16
	*StrainPhlan 3 df*	na	na	na	na	Na
	*StrainPhlan 3 new*	70	278	93%	57%	0.66
*S. aureus* (OP)	MetaPhlAn 3	74	131	98%	60%	0.74
	*StainPhlAn df*	40	134	100%	33%	0.49
	*StrainPhlan 3 new*	56	132	99%	46%	0.62
*M. catarrhalis* (NP)	MetaPhlAn 3	132	228	84%	89%	0.81
	Metagenome-Atlas	119	218	87%	85%	0.82
	*StrainGE*	53	241	96%	38%	0.52
	*StrainPhlan 3 df*	89	256	94%	60%	0.70
	*StrainPhlan 3 new*	119	243	89%	80%	0.80
*H. influenzae* (NP)	MetaPhlAn 3	152	205	79%	94%	0.83
	Metagenome-Atlas	150	182	77%	96%	0.84
	*StrainGE*	60	226	96%	38%	0.53
	*StrainPhlan 3 df*	na	na	na	na	na
	*StrainPhlan 3 new*	122	233	90%	75%	0.79
*H. influenzae* (OP)	MetaPhlAn 3	66	96	55%	80%	0.58
	*StrainPhlan 3 df*	na	na	na	na	na
	*StrainPhlan 3 new*	62	121	70%	75%	0.63

^*a*^ Data for selected bacterial species from NP and OP are indicated for default (df) and new settings (new). Culture results were considered as the “golden standard.” For certain settings, no values were achievable for the available data (na). Strain resolution tools are indicated in italic.

^*b*^ TP = True Positive; TN = True negative. See text for details.

### Adjusting StrainPhlAn 3 parameters for optimal species detection of low biomass samples

**Fig 1:**
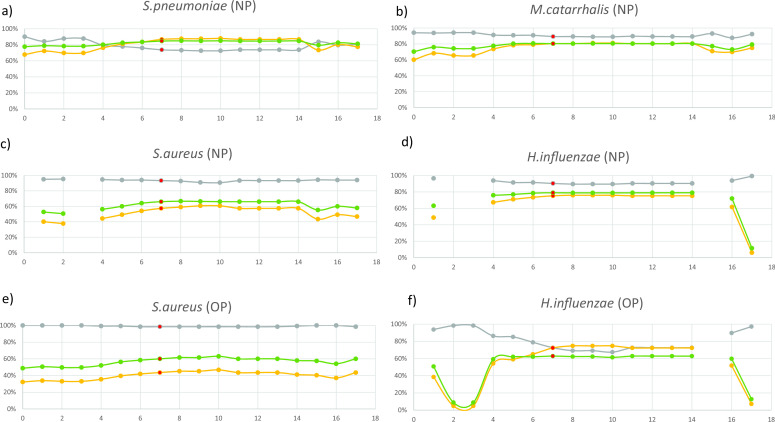
Benchmarking the different set of parameters. The default set of parameters is illustrated in run No. 0 and is BREADTH_THRESHOLD 80, TRIM_SEQUENCES 50, MARKER_IN_N_SAMPLES 80, and SAMPLE_WITH_N_MARKERS 20. Panels a, b, c, and d represent benchmarking for NP data; e and f for OP data. The default (No. 0) and 17 different sets of parameters (No. 1–17) are illustrated on the x-axis, and their characteristics are described in Table S1. Gray = specificity, yellow = sensitivity, and green = F1-score. The optimized sets of parameters are shown at No. 7 (indicated in red).

We next assessed how species detection performed when using higher coverage thresholds required by strain resolution tools StrainPhlAn 3 and StrainGE, comparing again the results to the culture data. Using default parameters, both tools showed low sensitivity (being less than 0.7) and F1 values for *Streptococcus pneumoniae*, *Moraxella catarrhalis*, *S. aureus*, and *Haemophilus influenzae* from NP samples (Table 2). However, StrainPhlAn performed better than StrainGE, presumably because, in contrast to StrainGE, it does not rely on an assembly expected to perform poor for low biomass samples. That is why, we focused our efforts on optimizing StrainPhlAn, hypothesizing that using less stringent parameters to further adapt for low coverage rates (and hence improving species detection), would not affect the performance of the tool for strain resolution. Benchmarking results of different parameter sets were received for the four (*S. pneumoniae*, *M. catarrhalis*, *S. aureus*, and *H. influenzae*) and the two species (*S. aureus* and *H. influenzae*) from the NP and OP data set, respectively (Fig. 1). The overall plot then suggested that the set of parameters No. 7 with a F1 value of 0.72 were the most optimal ones for further analysis (Fig. S2; Table S1). Table 2 shows the performance values for species detection using the newly set of parameters No. 7. Using these optimized parameters, we noticed that StrainPhlAn 3 analyses would also be feasible for frequently found commensal bacterial species in the NP data set, i.e., *Streptococcus mitis* (*n* = 281), *Dolosigranulum pigrum* (*n* = 240), and *Corynebacterium pseudodiphtheriticum* (*n* = 165) for which, however, no culture data were available to confirm the taxonomic assignment (Table S2).

### Assessment of StrainPhlAn 3 for species resolution

**Fig 2:**
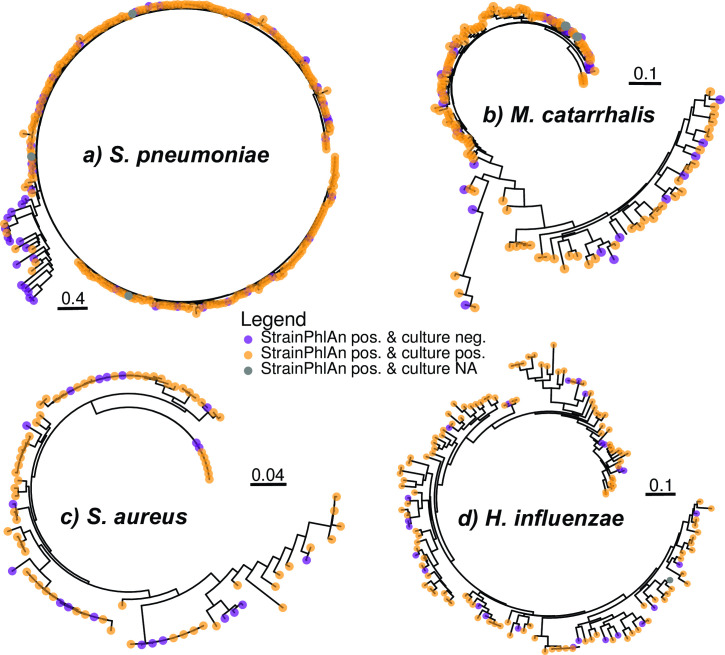
StrainPhlAn-derived trees from NP data sets. Trees are StrainPhAn 3 generated for (a) *S. pneumoniae*, (b) *M. catarrhalis,* (c) *S. aureus*. and (d) *H. influenzae* from NP data and were using the optimized parameters of StrainPhlAn 3. Samples are indicated in different colors depending on the presence/absence of culture data [i.e. purple = found in StrainPhlAn but not culture, yellow = found with StrainPhlAn and culture, and gray = found with StrainPhlAn but no culture data available (NA)].

To further investigate the limitations of the specificity and sensitivity of bacterial species captured by StrainPhlAn 3, we subsequently inspected trees generated by StrainPhlAn 3 for *S. pneumoniae*, *M. catarrhalis*, *S. aureus*, and *H. influenzae* (Fig. 2A through D). For *S. pneumoniae*, we identified a small, separate cluster of 22 samples for which a positive culture of *S. pneumoniae* was only found in six samples (Fig. 2A). To investigate the latter, we additionally added the streptococcal metagenome-assembled genomes (MAGs) from the Metagenome-Atlas analyses run on the same samples as reference genomes into the StrainPhlAn 3 tree. We found that the tree indeed split into *S. pneumoniae* and non-*S*. *pneumoniae* strains (Fig. S3). *S. pneumoniae* was also frequently detected in the OP samples by StrainPhlAn 3 (*n* = 246; 94.6%; Table S3) despite only a single *S. pneumoniae* culture was observed (*n* = 1; 0.4%). Taken together, these analyses suggest that StrainPhlAn occasionally misidentifies *S. pneumoniae*, although we cannot completely rule out, that few *S. pneumoniae* isolates were missed with the applied culture conditions. The StrainPhlAn 3 tree for *M. catarrhalis* also splits into two clusters, but in contrast to *S. pneumoniae*, both were readily detected through culture (Fig. 2B). No obvious clustering has been observed for *S. aureus* and *H. influenzae* and samples which were culture negative but species positive according to StrainlPhlAn 3 were found randomly all along the trees (Fig. 2C and D).

The low detection rates of *S. aureus* using StrainPhlAn 3 (Table 2) were also further investigated in the OP samples. We investigated if false positives/negatives were caused by a lower coverage, but we did not observe any difference in the mean bacterial reads (Fig. S4A). We also compared quantitative culture information (from 0 to 4 according to number of colonies during culturing) with StrainPhlAn 3 output (Fig. S4B). We found that the higher the quantity of *S. aureus* detected by culture, the more likely *S. aureus* was also detected by StrainPhlAn 3 (*P* < 0.01). This correlation was independent of the total number of (bacterial) reads, i.e., read coverage.

### Evaluating newly defined parameters of StrainPhlAn 3 for strain resolution

**Fig 3:**
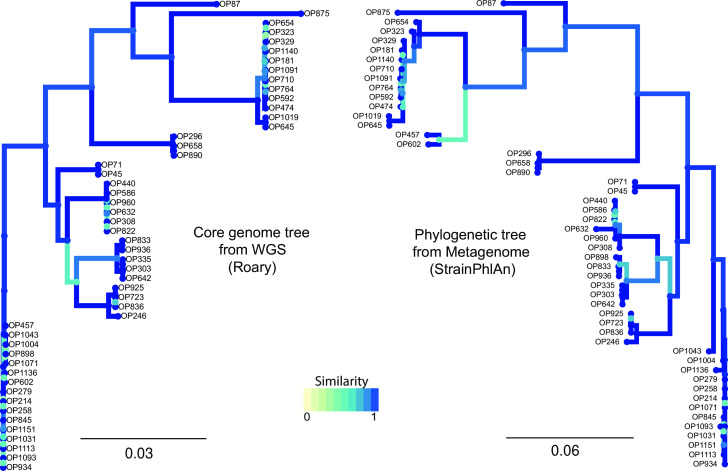
Phylogenetic trees of *S. aureus* from OP data sets. On the left, core genome tree from WGS data of *S. aureus* isolates. On the right, the phylogenetic tree was created from metagenomic data of OP samples using the adjusted parameters of StraiPhlAn. Sample IDs are indicated in black.

We next compared strain diversity of *S. aureus* captured by StrainPhlAn 3 to strain diversity represented by WGS of *S. aureus* isolated from OP samples by visualizing side by side the phylogenetic tree generated by each approach (Fig. 3). Despite using different data sources and analyses, both trees showed a strikingly congruent topology, with only three samples (OP457, OP602, and OP898) showing a difference in placement. This could be because the isolated strains differed from the most dominant one detected through StrainPhlAn 3.

We also investigated whether the adjusted parameters of StrainPhlAn 3 (which improved species detection) would diminish the performance of strain resolution as compared to the default parameters. Comparing StrainPhlAn 3 trees of *S. aureus* between default and adjusted parameters showed comparable results, indicating that our change of parameters did not impact strain resolution (Fig. S5).

To further investigate if our chosen parameters are too relaxed and, therefore, can produce low-quality phylogenetic results, we have also created the StrainPhlAn 3 trees of *M. catarrhalis* between default and adjusted parameters from the NP samples (Fig. S6). Again, the results were congruent indicating that our parameters are not too relaxed and still produce similar quality phylogenetic results as compared to the standard settings and, at the same time, improve species detection. However, careful benchmarking is generally recommended for other bacterial species and data from other studies if non-default parameters are to be used.

### Evaluating pairwise SNVs and strain retention in NP samples

**Fig 4:**
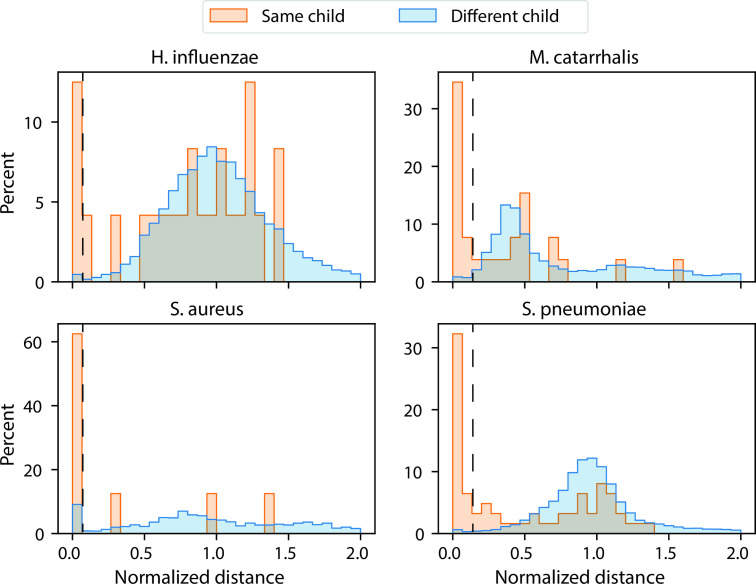
Strain retention analyses based on normalized genetic distances of four different species of the NP data set. The all-versus-all normalized genetic distances have been separately calculated for *S. pneumoniae, M. catarrhalis*, *H. influenzae*, and *S. aureus.* Values were binned in intervals of 0.1. Bins with values 0.0–0.1 (for *S. aureus* and *H. influenzae*) and 0.0–0.2 (for *S. pneumoniae* and *M. catarrhalis*) were defined for strain retention (dotted lines; see text for details).

Having defined the optimized parameters for StrainPhlAn 3 for our data sets, we next investigated strain retention in the NP samples which were collected at two different time points for each subject. We first generated pairwise single nucleotide variation (SNV) rates for *S. pneumoniae*, *M. catarrhalis*, *H. influenzae*, and *S. aureus* of the NP data set (Fig. S7). We then binned the normalized distance values into intervals of 0.1 and visualized the results in a linear and log-transformed manner (Fig. 4; Fig. S8 and S9). For different infants (inter-comparisons), we found that values from different timepoints were strikingly comparable to values from the same timepoints (Fig. S8).

However, as expected, the distance values were lower in samples from the same infant as compared to different infant (Fig. 4; Fig. S9). Therefore, frequent longitudinal carriage of identical isolates, i.e., strain retention can be assumed. Based on inter- vs intra-comparisons, we suggest defining bins with values 0.0–0.1 (for *S. aureus* and *H. influenzae*) and 0.0–0.2 (for *S. pneumoniae* and *M. catarrhalis*) for strain retention for the four species. Using these definitions, the values for strain retention were 40.8% (*S. pneumoniae*), 41.9% (*M. catarrhalis*) 16.7% (*H. influenzae*), and 55.6% (*S. aureus*; Fig. 4).

### Investigating strain retention of *S. aureus* over time in children with CF

**Fig 5:**
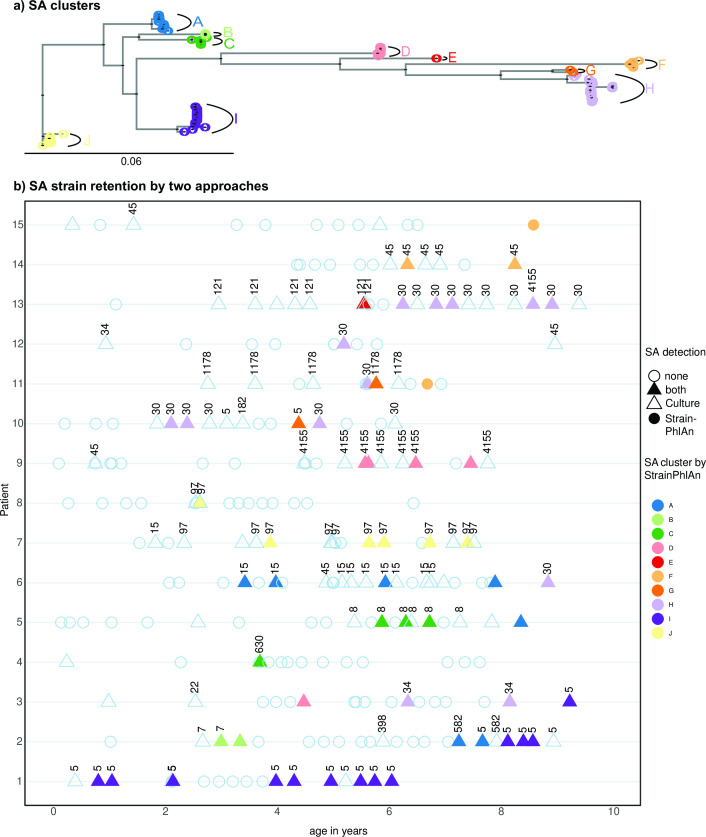
Clustering and longitudinal visualization of *S. aureus* strains of OP samples. Phylogenetic tree was created with StrainPhlAn using the new parameters from metagenomic data, highlighting 10 clusters (1-J) (Fig. 5A). Strain retention of *S. aureus* was investigated in a total of fifteen individuals with CF, sampled for up to the first 10 years of life (Fig. 5B). Strain retainment was investigated by (i) extracting the MLST from the WGS from *S. aureus* (SA) and (ii) using the clustering information from metagenomic data (shown as SA_clusters from A-J with different colors). For the detection of SA we used four categories. No *S. aureus* found by culture and metagenomic sequencing (indicated by an empty circle), *S. aureus* found by culture and metagenomic sequencing (filled triangle), *S. aureus* found by culture but not metagenomic sequencing (empty triangle), and *S. aureus* found by metagenomic sequencing but not by culture (filled circle). StrainPhlAn clusters are indicated with different colors. Numbers reflect the sequence types from MLST found in the respective *S. aureus* isolates. As for triangles without numbers, *S. aureus* by culture has been reported, but the isolate has not been kept for WGS.

Retaining of *S. aureus* strains over time was also investigated in the OP samples. Based on the StrainPhlAn tree of *S. aureus*, we found 10 clusters of samples which were subsequently used as information for the definition of strain retention in the OP samples (Fig. 5A). Figure 5B shows this cluster assignment within samples over time and, in addition, Multi-Locus Sequence Typing (MLST) data (extracted from WGS data from isolates genomes). Generally, we found that up to three strains were retained for the majority of patients as seen for both metagenomic clusters and MLST. In some cases, we observed that the same strain was retained for many years (ST97 and cluster J for subject ID7; ST5 and cluster I for subject ID1). This analysis shows that strain resolution derived from metagenomic data is at least comparable to the traditionally used MLST scheme (at least for *S. aureus*).

## DISCUSSION

Bacterial strain characterization is indispensable for, in particular, clinically relevant bacteria also called pathobionts (19). It is classically achieved by bacterial culture and subsequent genomic characterization. This is cumbersome if many members of the microbiome are to be characterized, and/or large sample sets are included. Metagenomic sequencing has the potential to provide bacterial strain resolution of multiple organisms at once. However, airway microbiome is low biomass, making metagenomic analysis a challenge and careful benchmarking necessary. In this study, using shotgun metagenomics, culture, and genomic data from OP and NP samples, we optimized species and strain resolution, enabling strain retention analyses in our longitudinal samples.

### Despite low microbial biomass, metagenomics allows reliable species detection

Using NP and OP samples from infants and children, we first confirmed that the samples were highly dominated by host DNA despite high overall read counts. We therefore expected limitations in reaching high sensitivity for the detection of bacterial species and strain resolutions in metagenomic data. This problem is well recognized, and alternative strategies like different DNA extraction techniques (20) or the Plate coverage algorithm have been suggested (21). Despite this potential issue, sensitivity, and specificity in detecting *S. pneumoniae*, *M. catarrhalis* and *H. influenzae* at the species level were quite good for both MetaPhlAn 3 and MAGs-based Metagenome-Atlas, compared to culture data.

### Strain resolution tools require optimization for species detection first

Ultimately, the goal of the study was to investigate bacterial strain retention. To this end, we assessed strain-level resolution tools such as StrainGE and StrainPhlAn 3 (13, 14). The first step was to assess whether the species assignation of these tools was performed correctly. We showed that performance using default parameters was insufficient since they require a higher sequencing coverage of the target genomes, this being especially limiting for assembly-based tools.

Despite these improvements, challenges still remained in the correct species detection. Reference-based tools such as StrainPhlAn reflect the currently accepted taxonomic structures and their inherent discrepancies. For example, despite being classified into several distinct species, *S. mitis*-like bacteria (including *S. pneumoniae*) are highly related (22) with homologous housekeeping genes (23), which constitutes a challenge for proper classification. Therefore, it comes as no surprise that we detected a number of samples as positive for *S. pneumoniae* using the StrainPhlAn 3 pipeline, while most likely, these samples contained other *S. mitis*-like strains. This problem is even more accentuated in our OP samples, and we therefore recommend to critically assess metagenomics-derived results for the identification of *S. pneumoniae* in OP samples. Of note, unlike the NP, the challenges of using OP samples to measure pneumococcal carriage are known (24, 25).

Another example, it has been described that there are seroresistant and serosensitive *M. catarrhalis* with separate evolutionary histories (26, 27). In our NP data set, this is reflected as two separate clusters indicating that *M. catarrhalis* should probably better be considered as two different subspecies. In addition, we also obtained somewhat low sensitivity values for *S. aureus.* This is not explained by the difficulties in the taxonomic species assignment using StrainPhlAn but rather by the rigidity of the cell wall of *S. aureus*. To achieve higher sensitivity values of *S. aureus*, a more stringent DNA extraction procedure would be needed (28) which may, however, lead to DNA loss for other species.

### Strain resolution is challenging with low genomic coverage but achievable with adequate tools optimization

Systematically capturing whole microbiome diversity, especially at the strain level is challenging for such low biomass samples, whether through metagenomics or classical approach, i.e., isolation and genotyping of the isolates (13). Nonetheless, analyses targeting specific pathobionts are highly relevant in a clinical context as information from pathobionts in combination with basic information from commensal bacteria could prove sufficient to address a number of clinical research questions (19). Being able to reconstruct full genomes from metagenomic data sets would allow efficient tracking of co-occurring strains with dedicated tools such as StrainGE. However, our study showed the limitations of such an approach in a low biomass setting context, where low genomic coverage does not allow the reconstruction of metagenomic assemblies with high enough quality for strain resolution. Detecting only the dominant strain of a sample is often considered a limitation of StrainPhlAn, but despite this, StrainPhlAn permitted strain retention analyses once the parameters were carefully optimized.

### Strain resolution allows longitudinal strain tracking

Strain identification and retention analyses are very important, especially in studies with longitudinal design (29). It allows the calculation of carriage duration of distinct strains and to study their association with health and disease. Though there are only two timepoints in the NP data set, strain retention, e.g., *S. pneumoniae*, was found to be 40.8% which is in a similar range as found in other studies using the same age (2 and 4 months of age) and time distance between samples (2 months; 29, 30).

In chronic diseases like CF, the ability to perform strain-resolved analyses in longitudinally collected samples may help identifying strains that are associated with disease exacerbation. In our study, we detected considerable long-term colonization of distinct *S. aureus* strains in children with CF. Though the sensitivity of the detection of *S. aureus* with the metagenomic data is somewhat reduced in some samples, the fact that the CF patients were sampled every 2–3 months for up to 10 years allowed to thoroughly assess strain retention even if one or the other sample was found to be negative (based on culture and/or metagenomic sequencing). In the future, this allows more in-depth investigation with clinical relevance such as on the consequences of antibiotic treatment on the strain retention, e.g., comparing recolonization vs strain retention.

### Strengths and limitations

The strength of this study is the parallel investigation of culture, whole genome, and metagenomic data of two different sets of samples. While many benchmarking studies are focusing on simulations and metanalyses of already existing data, comparisons of culture and metagenomic data are rather rare. Bacterial cultures are not only considered as the golden standard for species assignation; successful culture demonstrates that metagenomic reads originate from viable bacterial cells. However, this study has some limitations. We only cultured four and two species from the NP and OP samples, respectively. Also, we only sequenced *S. aureus* isolates from the OP samples as isolates from the NP samples were not available for sequencing. Finally, we have not analyzed data from the lower airway microbiome which has been shown to be clinically relevant but not easy to retrieve due to the invasive nature of sampling (5). However, it has also been shown that the lower airways are seeded by both the OP and NP microbiome (17) which were included in our study. Finally, the StrainPhlAn pipeline enables strain retention analyses but does not allow the analyses for structural genome variations including, e.g., the genomic insertion and loss of phages typically associated with shifts of virulence and antimicrobial susceptibility in the airways of CF patients

### Conclusions

We have performed benchmarking analyses for StrainPhlAn and provided strain retention results for two different metagenomic data sets from the upper respiratory tract. However, while shotgun metagenomic data analysis remains challenging for low biomass samples, it allows for strain-level resolution in contrast to 16S rRNA gene sequencing (1). Strain level information is indispensable to associate the presence of microbial strains or subclades of microbial species with disease phenotypes, but a careful evaluation of tools and parameters is a prerequisite, especially for low biomass samples (16).

## MATERIALS AND METHODS

### Characteristics of the data sets

We reanalyzed previously published shotgun metagenomic data from NP samples of the Microbiota and Health study from infants conducted in Dhaka, Bangladesh (18). The study design has been described and included the collection of NP samples from 267 infants at bi-monthly intervals during scheduled visits (18). Therefore, data were available for infants being asymptomatic for respiratory infections at 2 and 4 months of age. NP samples were initially stored at −20°C but then transferred to −80°C for shipment until further analysis (31). In total, an average of 38.1 million (M) read pairs per sample was re-processed for 422 samples. Culture data for *H. influenzae*, *S. aureus*, *S. pneumoniae*, and *M. catarrhalis* were available for all samples (31).

OP samples were obtained from a prospective follow-up study of infants diagnosed with CF by newborn screening at the university hospital (Inselspital) in Bern, Switzerland (32, 33). Ethical approval has been obtained (KEK-ethics no. 114/11). For this project, OP samples from 15 children who were followed up until 10 years of age were included. OP swabs were collected approximately every 3 months during clinical visits and, upon arrival, kept at −80°C until DNA extraction was done (see below). In parallel, routine culturing of each OP sample was performed during which a broad range of “clinical relevant” bacterial species were received. In brief, OP swabs from CF patients were streaked out onto Columbia sheep blood agar, which was used for the cultivation of non-fastidious and fastidious microbes as a screen for overall growth (34). In addition, MacConkey Agar was used for the selection of Gram-negative bacteria (i.e., *P. aeruginosa*). Most importantly for this study, Chocolate agar with the addition of bacitracin and Mannitol salt agar plates were used for the investigation of *H. influenzae* and *S. aureus*, respectively. Species identification was done using MALDI-TOF mass spectrometry analysis and Antibiogram and semi-quantitative information of bacterial growth from all isolates were also reported. In this study, we focused on culture results from *H. influenzae* and *S. aureus* due to high numbers in the OP swabs. Also, isolates identified as *S. aureus* were picked and kept at −80°C until further (WGS) analysis (see below for details).

### Sample preparation and sequencing of the OP microbiome

OP swabs were swiped in a 1.5 mL tube containing 500 µL sterile phosphate-buffered saline (PBS). 200 µL of the bacterial suspension was used for DNA extraction using Qiacube running the DNA Mini extraction program. The shotgun metagenomic sequencing of the OP microbiome was done with a NovaSeq 6000 PE150 yielding approximately 10 G of raw data per sample. A negative template control sample was also included but yielded insufficient read quality.

### Processing of metagenomic reads

For the present study, shotgun metagenomic reads from both data sets were analyzed. Human reads were removed in two runs of BBmap v38.84 (35): a first coarse run to remove a majority of human reads (parameters: mode fast = true, minratio = 0.9; maxindel = 3, minhits = 2, kmer length = 14). The second run used default parameters for more sensitivity in removing remaining human reads. Moreover, all reads mapping to *Escherichia coli* from NP data set were not considered for this study following recommendations of the original analysis publication (18). The quality of the reads was checked with FastQC (36) and MultiQC (37).

### Taxonomic profiling through MetaPhlAn 3 and MAGs

Taxonomic profiling was done with MetaPhlAn 3 using the default parameters (38). In addition, the shotgun metagenomic data from the NP samples was re-analyzed using Metagenome-Atlas v 2.12 (38) to generate MAGs. In short, using tools from the BBMap suite v39 (35), reads were quality trimmed, and contaminations from the human genome were filtered out. Reads were error corrected and merged before assembly with metaSPAdes v3.15 (39). Contigs were binned using MetaBAT v2.15 (40) and MaxBin v2.2 (41), and their predictions were combined using DAS Tool v1.1 (42). The predicted MAGs, which had at least 50% completeness and <10% contamination based on the estimation by CheckM v1.1 (43), were clustered at 95% average nucleotide identity using dRep v3.2. The resulting 71 species representative MAGs were taxonomically annotated with GTDB-tk v2.1 to the Genome Taxonomy Data Base release 207 (44) and quantified using by the median of coverage in 1 kb windows along the genomes. A genome was called present if the median abundance was larger than 0. Reads were mapped using Minimap v2.24 (45).

### Strain resolution analyses

Based on MAGs, strain inference was attempted using StrainGE v.1.3.3 (14) according to the documentation. In brief, the reads were mapped using bwa mem2 (46) to the detected strains starting from all complete RefSeq genomes from NCBI GenBank of the genera *Streptococcus* (*n* = 536; accessed on 24 October 2022), *Staphylococcus* (*n* = 317; accessed on 24 October 2022), *Moraxella* (*n* = 31; accessed on 20 December 2022), and *Haemophilus* (*n* = 87; accessed on 20 December 2022). The recommended threshold of 0.5 coverage and the detection using *k*-mers were used as the detection limit.

Based on MetaPhlAn 3 output, StrainPhlAn 3 was run with different sets of parameters for benchmarking (38). In total, four parameters can be changed: BREADTH_THRESHOLD, TRIM_SEQUENCES, MARKER_IN_N_SAMPLES, and SAMPLE_WITH_N_MARKERS. BREADTH_THRESHOLD describes the breadth of coverage threshold for the consensus markers and is used on the step of creating a marker file for each sample. The default value is 80%, and we used the range from 20% to 80%. TRIM_SEQUENCES sets the number of bases to be removed from both ends of the marker. The default value is 50, and we checked for the range from 30 to 50. This parameter was kept default, as the change in this parameter didn’t have an impact on the results. MARKER_IN_N_SAMPLES defines how many samples should have a marker so that it is kept for the analysis. The default value is 80%, and it should be lowered if species of interest are not expected to be found in less than 80% of the samples. SAMPLE_WITH_N_MARKERS defines the minimum number of markers to keep a sample in the analysis. The default value is 20, but some species have less markers available in the database (e.g., *H. influenzae* for which there were only 15 markers available in the database). Therefore, we investigated the performance of StrainPhlAn 3. Altogether, the default and 17 different sets of parameters were assessed, and the analyses were individually performed for four (*S. pneumoniae*, *M. catarrhalis*, *H. influenzae*, and *S. aureus)* and two (*H. influenzae* and *S. aureus)* species from the NP and OP data set, respectively. These species were chosen as the culture data were available for them. Also, an overall plot for all the bacterial species was created, and adjusted parameters were finally chosen based on the highest F1 value while being closest to default parameters (therefore, we would not expect a major impact on strain resolution). The full set of parameters can be seen in the Table S1. Using the adjusted parameters, phylogenetic trees were produced by StrainPhlAn3 based on a comparison of marker genes.

### Whole genome shotgun analysis of from OP samples and phylogenetic comparison to StrainPhlAn 3 marker genes

To compare metagenomic-based phylogenetic resolution with genomic-based phylogenetic resolution, we used the WGS of *S. aureus* isolates from the OP samples. In brief, *S. aureus* cells were added into 200 µL PrepMan Ultra reagent in a microcentrifuge tube. Cells were then vortexed and boiled for 10 min at 100°C. Subsequently, we centrifuged at 8,500 × *g* for 3 min and transferred the supernatant to a clean 1.5 mL Eppendorf tube. The sequencing was done with a NovaSeq PE150. The microbial whole genome library preparation was performed for 350 bp. As for the Q30 of the PE150, this was indicated as Q30 >80%, and 1 G of raw data per *S. aureus* isolate was received. The quality of the reads was assessed with FastQC and MultiQC, and assembly was performed with SPAdes v3.15.2 (47) with default parameters. The core genome tree was created with Roary v3.11.0 (48) and visualized next to the StrainPhlAn3 tree using phylo.io (48). For this visualization, 50 OP samples were included for which both metagenomic and whole genome data were available for *S. aureus*.

### Strain retention analyses in the NP data set

In order to trace strain retention, we first calculated the pairwise SNV rates using PhyloPhlAn integrated into StrainPhlAn. In brief, PhyloPhlAn computes the SNV rates as the number of bases that differ between each pair of samples (using the alignment of used marker genes) divided by the number of positions shared (i.e., containing no gaps) between the samples. Subsequently, the normalized distances were calculated from the SNV rate for each species by normalizing the values with the median of the SNV rate. The all-versus-all normalized genetic distances were generated for the four bacterial species (*S. pneumoniae*, *M. catarrhalis*, *H. influenzae*, and *S. aureus)* from the NP data set and grouped according to three different categories: comparing different timepoints for different infants (inter-comparison), same timepoint for different infants (inter-comparison), and different timepoints for same infants (intra-comparison). Values were binned into intervals of 0.1 in a histogram. Bins with values 0.0–0.1 (for *S. aureus* and *H. influenzae*) and 0.0–0.2 (for *S. pneumoniae* and *M. catarrhalis*) were defined for strain retention. These values were chosen based on the comparisons of within with between values with the latter being low as a condition.

### Strain retention analyses in the OP data set

To analyze strain retention of *S. aureus* in the OP data set, a tree of shotgun metagenomic data were first created by StrainPhlAn and again visualized with phylo.io (48). Clusters of strains were then assigned based on the tree, using a threshold 0.01 branch length. The MLST profiles were extracted from the assembled *S. aureus* genomes using the center for genomic epidemiology website (https://cge.food.dtu.dk/services/MLST/). The resulting MLSTs were used for strain retention analyses of the *S. aureus* isolates. Strain clusters were longitudinally plotted together with the ST types of *S. aureus* to allow direct comparison of the two approaches for their potential to determine strain retention.

## Data Availability

All raw Illumina sequencing reads for the whole genome sequencing data were deposited in the European Nucleotide Archive (ENA) under study accession number PRJNA930445. All raw Illumina sequencing reads from the shotgun metagenomic sequencing runs (OP data) were deposited in the European Nucleotide Archive (ENA) under study accession number PRJNA931830.

## SUPPLEMENTAL MATERIAL

Supplemental figures msystems.00724-23-s0001.docx Figures S1 to S9.

Supplemental tables S1 to S3 msystems.00724-23-s0002.docx Tables S1 to S3.

ASM does not own the copyrights to Supplemental Material that may be linked to, or accessed through, an article. The authors have granted ASM a non-exclusive, world-wide license to publish the Supplemental Material files. Please contact the corresponding author directly for reuse.
