# Supplementary material for: Informed interpretation of metagenomic data by StrainPhlAn enables strain retention analyses of the upper airway microbiome
Source: mSystems. 2023 Nov 2;8(6):e00724-23. doi: 10.1128/msystems.00724-23 (PMC10734448; doi:10.1128/msystems.00724-23)
Supplement: Supplemental figures — Figures S1 to S9. [file msystems.00724-23-s0001.docx]

**Supplementary figures**


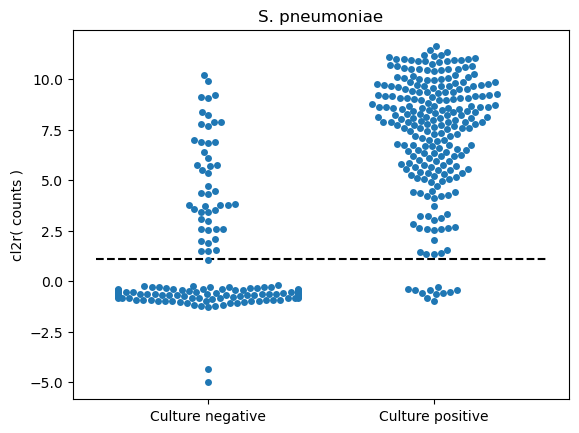

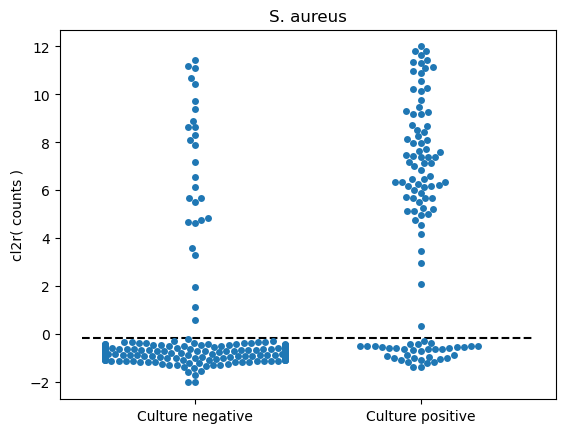

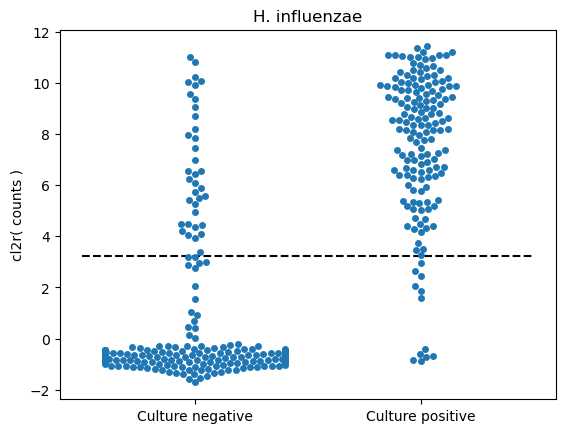

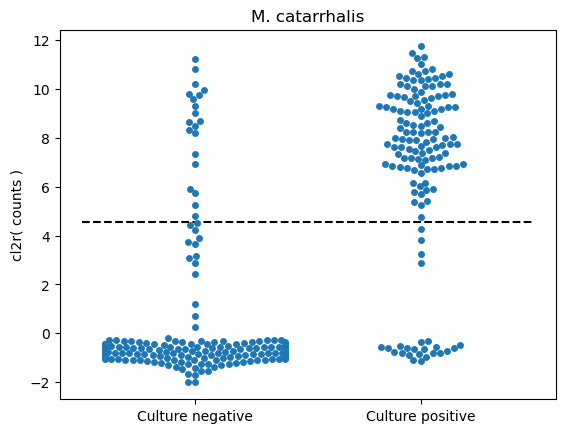


**Fig. S1: Benchmarking parameters for species detection using metagenome-atlas.** Illustrated are the 'best' cut offs for four different bacterial species.

**Fig. S2: Benchmarking the different set of parameters (summary plot).** The default set of parameters is illustrated in try No. 0 and is BREADTH_THRESHOLD 80, TRIM_SEQUENCES 50, MARKER_IN_N_SAMPLES 80 and SAMPLE_WITH_N_MARKERS 20. This is a summary plot of the panels a-f from Figure 1. The different tries (0-17) are illustrated on the x-axis and their characteristics are described in Supplementary table 1. Grey; Specificity, Yellow; Sensitivity and Green; F1 score. Default and chosen settings are shown at try '0' and at '7' (indicated in red), respectively.

**Fig. S3: StrainPhlAn based tree for *Streptococcus pneumoniae* from NP data.** Metagenomic assemblies (MAGs) are indicated in different cultures (red).

**Fig. S4A:** Correlation of bacterial reads with culture results. Correlations are indicated according to true positive (TP), false positive (FP), true negative (TN) and true positive (TP) of *S. aureus* using culture as the gold standard*;* mean values are shown*.* **Fig. S4B**; correlation plot. Shown are quantitative culture information (from 0 to 4) according to number of colonies during culturing correleated with StrainPhlAn 3 output; mean values are shown

**Fig. S5: Phylogenetic trees of *S. aureus* from OP data.** Phylogenetic trees were created with StraiPhlAn from metagenomic and whole genome sequencing (WGS) data. On the left and on the right side are illustrated the trees using the default and optimised settings, respectively.

**Fig. S6: Phylogenetic trees of *M. catarrhalis*, from NP data.** Phylogenetic trees were created with StraiPhlAn from metagenomic data. On the left and on the right side are illustrated the trees using the default and optimised settings, respectively.

**Fig. S7: Pairwise single nucleotide variations (SNVs) for four different bacterial species of the NP**

Mutation rates are shown for *S. pneumoniae,* *M. catarrhalis*, *H. influenzae* and *S. aureus*. Values represent samples from different children at different months (red), samples from same child (green; at 2 and 4 months) and from different children at the same age.

**Fig. S8: Strain retention analyses based on normalized genetic distances of four different species of the NP** (Different child (same month) versus different child) (different month)

The all-versus-all normalized genetic distances have been separately calculated for *S. pneumoniae,* *M. catarrhalis*, *H. influenzae* and *S. aureus.* Values were binned in intervals of 0.1 and then log-transformed.

**Fig. S9: Strain retention analyses based on normalized genetic distances of four different species of the NP** (Same child versus different child)

The all-versus-all normalized genetic distances have been separately calculated for *S. pneumoniae,* *M. catarrhalis*, *H. influenzae* and *S. aureus.* Values were binned in intervals of 0.1 and then log-transformed.
