# Supplementary material for: Informed interpretation of metagenomic data by StrainPhlAn enables strain retention analyses of the upper airway microbiome
Source: mSystems. 2023 Nov 2;8(6):e00724-23. doi: 10.1128/msystems.00724-23 (PMC10734448; doi:10.1128/msystems.00724-23)
Supplement: Supplemental tables S1 to S3 — Tables S1 to S3. [file msystems.00724-23-s0002.docx]

**SUPPLEMENTARY Tables**

**Supplementary table 1: Parameters according to 18 different tries***

|  | |  |  | |  | |
| --- | --- | --- | --- | --- | --- | --- |
|  | |  |  | |  | |
| Try | BREADTH_  THRESHOLD | TRIM_  SEQUENCES | MARKER_IN_N_  SAMPLES | SAMPLE_WITH_N_  MARKERS | |  |
| 0 | 80 | 50 | 80 | 20 | |  |
| 1 | 80 | 50 | 50 | 10 | |  |
| 2 | 80 | 50 | 50 | 15 | |  |
| 3 | 80 | 50 | 80 | 15 | |  |
| 4 | 80 | 50 | 50 | 5 | |  |
| 5 | 70 | 50 | 50 | 5 | |  |
| 6 | 60 | 50 | 50 | 5 | |  |
| 7 | 50 | 50 | 50 | 5 | |  |
| 8 | 40 | 50 | 50 | 5 | |  |
| 9 | 30 | 50 | 50 | 5 | |  |
| 10 | 20 | 50 | 50 | 5 | |  |
| 11 | 50 | 50 | 40 | 5 | |  |
| 12 | 50 | 50 | 30 | 5 | |  |
| 13 | 50 | 40 | 50 | 5 | |  |
| 14 | 50 | 30 | 50 | 5 | |  |
| 15 | 50 | 50 | 50 | 20 | |  |
| 16 | 50 | 50 | 50 | 10 | |  |
| 17 | 50 | 50 | 50 | 15 | |  |

*Default and chosen settings are defined in try 0 and 7 (yellow), respectively

Supplementary table 2: Species found by StrainPhlAn in NP samples (new parameters used)

| **Species** | **Found in # samples** |
| --- | --- |
| Escherichia coli - contaminant | 397 |
| Streptococcus mitis | 281 |
| Streptococcus pneumoniae | 263 |
| Dolosigranulum pigrum | 240 |
| Streptococcus sp M334 | 230 |
| Corynebacterium pseudodiphtheriticum | 165 |
| Haemophilus haemolyticus | 152 |
| Moraxella catarrhalis | 150 |
| Haemophilus influenzae | 148 |
| Corynebacterium accolens | 148 |
| Haemophilus sp C1 | 142 |
| Streptococcus oralis | 126 |
| Streptococcus sp SK643 | 118 |
| Ochrobactrum sp POC9 | 102 |
| Streptococcus sp HMSC070B10 | 96 |
| Staphylococcus aureus | 90 |
| Human betaherpesvirus 5 | 88 |
| Staphylococcus epidermidis | 73 |
| Moraxella nonliquefaciens | 69 |
| Moraxella lacunata | 58 |
| Streptococcus phage MM1 | 51 |
| Acinetobacter pittii | 50 |
| Moraxella osloensis | 44 |
| Streptococcus sp NLAE zl C503 | 42 |
| Thermus parvatiensis | 36 |
| Thermus thermophilus | 36 |
| Moraxella lincolnii | 33 |
| Enhydrobacter aerosaccus | 31 |
| Staphylococcus haemolyticus | 30 |
| Haemophilus aegyptius | 26 |
| Streptococcus phage PH10 | 26 |
| Acinetobacter sp WC 141 | 24 |
| Acinetobacter baumannii | 22 |
| Acinetobacter phage Bphi B1251 | 21 |
| Streptococcus sp oral taxon 058 | 20 |
| Bacillus cereus group | 20 |
| Cutibacterium acnes | 16 |
| Staphylococcus argenteus | 15 |
| Moraxella equi | 15 |
| Staphylococcus phage PT1028 | 14 |
| Acinetobacter nosocomialis | 13 |
| Staphylococcus capitis | 13 |
| Staphylococcus arlettae | 12 |
| Staphylococcus phage StB20 | 12 |
| Staphylococcus sciuri | 12 |
| Sphingomonas paucimobilis | 11 |
| Lysinibacillus sphaericus | 11 |
| Lactococcus lactis | 10 |
| Comamonas aquatica | 10 |
| Streptococcus pseudopneumoniae | 9 |
| Klebsiella pneumoniae | 9 |
| Klebsiella quasipneumoniae | 8 |
| Sphingobium yanoikuyae | 8 |
| Aerococcus viridans | 8 |
| Haemophilus sp HMSC71H05 | 7 |
| Paracoccus sanguinis | 7 |
| Staphylococcus hominis | 7 |
| Staphylococcus saprophyticus | 7 |
| Malassezia restricta | 6 |
| Gemella haemolysans | 6 |
| Staphylococcus caprae | 6 |
| Lysinibacillus fusiformis | 6 |
| Pseudomonas aeruginosa group | 6 |
| Enterobacter cloacae complex | 6 |
| Klebsiella variicola | 5 |
| Streptococcus salivarius CAG 79 | 5 |
| Acinetobacter schindleri | 5 |
| Staphylococcus virus PH15 | 5 |
| Bacillus oceanisediminis | 5 |
| Sphingobium lucknowense | 5 |
| Staphylococcus virus CNPH82 | 5 |
| Acinetobacter variabilis | 5 |
| Pseudomonas putida group | 5 |
| Aerococcus urinaeequi | 5 |
| Bifidobacterium longum | 4 |
| Sphingobacterium multivorum | 4 |
| Brevundimonas diminuta | 4 |
| Streptococcus parasanguinis | 4 |
| Staphylococcus xylosus | 4 |
| Klebsiella quasivariicola | 4 |
| Actinobacillus ureae | 4 |
| Streptococcus salivarius | 4 |
| Elizabethkingia anophelis | 4 |
| Lactobacillus gasseri | 4 |
| Lactobacillus paragasseri | 4 |
| Staphylococcus gallinarum | 4 |

Supplementary table 3: Species found by StrainPhlAn in OP samples

| **Species** | **Found in # samples** |
| --- | --- |
| Streptococcus mitis | 252 |
| Streptococcus sp M334 | 250 |
| Streptococcus pneumoniae | 246 |
| Streptococcus oralis | 238 |
| Streptococcus sp SK643 | 236 |
| Streptococcus sp HMSC071D03 | 233 |
| Rothia mucilaginosa | 231 |
| Neisseria subflava | 228 |
| Haemophilus sp HMSC71H05 | 227 |
| Streptococcus infantis | 227 |
| Streptococcus sp HMSC070B10 | 225 |
| Streptococcus sp HMSC034E03 | 223 |
| Granulicatella elegans | 221 |
| Neisseria flavescens | 221 |
| Neisseria polysaccharea | 220 |
| Haemophilus parainfluenzae | 218 |
| Streptococcus sp oral taxon 058 | 217 |
| Haemophilus haemolyticus | 211 |
| Streptococcus parasanguinis | 210 |
| Neisseria gonorrhoeae | 210 |
| Haemophilus sp C1 | 209 |
| Neisseria meningitidis | 207 |
| Streptococcus sp HMSC067H01 | 204 |
| Gemella haemolysans | 203 |
| Veillonella infantium | 201 |
| Prevotella melaninogenica | 201 |
| Streptococcus salivarius | 198 |
| Neisseria sp HMSC064E01 | 193 |
| Streptococcus salivarius CAG 79 | 192 |
| Fusobacterium sp oral taxon 370 | 191 |
| Streptococcus sp F0442 | 191 |
| Actinomyces odontolyticus | 190 |
| Aggregatibacter sp oral taxon 458 | 189 |
| Streptococcus peroris | 187 |
| Streptococcus australis | 185 |
| Streptococcus sp HPH0090 | 183 |
| Porphyromonas somerae | 182 |
| Neisseria macacae | 180 |
| Veillonella sp T11011 6 | 178 |
| Streptococcus vestibularis | 176 |
| Prevotella sp F0091 | 175 |
| Veillonella atypica | 174 |
| Actinomyces sp HMSC035G02 | 171 |
| Neisseria sicca | 171 |
| Prevotella sp oral taxon 306 | 171 |
| Fusobacterium periodonticum | 171 |
| Prevotella jejuni | 169 |
| Streptococcus thermophilus | 169 |
| Actinomyces sp HPA0247 | 163 |
| Campylobacter concisus | 160 |
| Fusobacterium nucleatum | 155 |
| Veillonella dispar | 154 |
| Neisseria perflava | 149 |
| Streptococcus phage EJ 1 | 146 |
| Prevotella scopos | 145 |
| Streptococcus phage PH10 | 144 |
| Actinomyces sp ICM47 | 143 |
| Gemella sanguinis | 142 |
| Haemophilus paraphrohaemolyticus | 137 |
| Neisseria mucosa | 130 |
| Leptotrichia sp oral taxon 215 | 126 |
| Streptococcus sp A12 | 124 |
| Neisseria lactamica | 123 |
| Veillonella parvula | 121 |
| Morococcus cerebrosus | 112 |
| Gemella morbillorum | 112 |
| Streptococcus phage SM1 | 111 |
| Leptotrichia hofstadii | 110 |
| Prevotella sp oral taxon 299 | 109 |
| Haemophilus influenzae | 109 |
| Prevotella pallens | 106 |
| Haemophilus parahaemolyticus | 106 |
| Leptotrichia wadei | 104 |
| Actinomyces sp oral taxon 180 | 98 |
| Leptotrichia sp oral taxon 212 | 98 |
| Atopobium parvulum | 96 |
| Haemophilus sputorum | 95 |
| Actinomyces graevenitzii | 95 |
| Prevotella salivae | 93 |
| Streptococcus pseudopneumoniae | 92 |
| Prevotella nigrescens | 92 |
| Neisseria sp oral taxon 014 | 92 |
| Oribacterium sinus | 91 |
| Neisseria cinerea | 89 |
| Prevotella sp oral taxon 473 | 87 |
| Capnocytophaga sputigena | 87 |
| Capnocytophaga gingivalis | 82 |
| Bulleidia extructa | 80 |
| Capnocytophaga leadbetteri | 73 |
| Actinomyces sp oral taxon 181 | 73 |
| Streptococcus sanguinis | 70 |
| Prevotella histicola | 70 |
| Ochrobactrum sp POC9 | 66 |
| Leptotrichia sp oral taxon 879 | 65 |
| Solobacterium moorei | 62 |
| Atopobium rimae | 61 |
| Leptotrichia sp oral taxon 498 | 61 |
| Leptotrichia sp oral taxon 847 | 60 |
| Eubacterium sulci | 58 |
| Lachnoanaerobaculum saburreum | 58 |
| Staphylococcus aureus | 56 |
| Prevotella shahii | 55 |
| Megasphaera micronuciformis | 54 |
| Mogibacterium diversum | 53 |
| Streptococcus phage MM1 | 53 |
| Fusobacterium hwasookii | 53 |
| Campylobacter mucosalis | 52 |
| Lautropia mirabilis | 51 |
| Veillonella tobetsuensis | 49 |
| Porphyromonas catoniae | 49 |
| Neisseria elongata | 49 |
| Campylobacter showae | 49 |
| Aggregatibacter aphrophilus | 48 |
| Streptococcus sp oral taxon 056 | 45 |
| Stomatobaculum longum | 45 |
| Tannerella sp oral taxon HOT 286 | 43 |
| Leptotrichia buccalis | 42 |
| Capnocytophaga sp oral taxon 338 | 41 |
| Capnocytophaga ochracea | 40 |
| Granulicatella adiacens | 39 |
| Prevotella oulorum | 38 |
| Moraxella nonliquefaciens | 38 |
| Moraxella catarrhalis | 34 |
| Eubacterium infirmum | 34 |
| Capnocytophaga sp oral taxon 863 | 33 |
| Phyllobacterium myrsinacearum | 33 |
| Oribacterium parvum | 32 |
| Eikenella corrodens | 29 |
| Streptococcus anginosus group | 28 |
| Peptostreptococcus stomatis | 25 |
| Oribacterium asaccharolyticum | 25 |
| Haemophilus pittmaniae | 24 |
| Veillonella rogosae | 24 |
| Moraxella lacunata | 24 |
| Kingella denitrificans | 23 |
| Actinomyces oris | 23 |
| Prevotella intermedia | 21 |
| Parvimonas sp oral taxon 110 | 20 |
| Campylobacter rectus | 20 |
| Escherichia coli | 19 |
| Capnocytophaga sp oral taxon 878 | 18 |
| Parvimonas sp oral taxon 393 | 18 |
| Staphylococcus phage PT1028 | 17 |
| Tannerella sp oral taxon 808 | 17 |
| Parvimonas micra | 17 |
| Pseudomonas aeruginosa group | 17 |
| Klebsiella pneumoniae | 16 |
| Enterobacter cloacae complex | 16 |
| Actinomyces naeslundii | 15 |
| Mogibacterium pumilum | 14 |
| Staphylococcus argenteus | 13 |
| Porphyromonas sp oral taxon 278 | 13 |
| Selenomonas sputigena | 13 |
| Actinomyces viscosus | 13 |
| Selenomonas flueggei | 13 |
| Pseudomonas phage F10 | 13 |
| Actinomyces johnsonii | 12 |
| Escherichia phage HK639 | 12 |
| Moraxella lincolnii | 12 |
| Stx2 converting phage 1717 | 12 |
| Alloprevotella rava | 11 |
| Prevotella loescheii | 11 |
| Lachnospiraceae bacterium oral taxon 096 | 11 |
| Cardiobacterium hominis | 11 |
| Kingella oralis | 11 |
| Treponema sp OMZ 838 | 11 |
| Pseudomonas phage phi297 | 11 |
| Abiotrophia defectiva | 10 |
| Mogibacterium timidum | 10 |
| Pseudomonas phage B3 | 10 |
| Pseudomonas virus Pf1 | 10 |
| Cardiobacterium valvarum | 9 |
| Actinomyces sp oral taxon 170 | 9 |
| Streptococcus virus Sfi21 | 8 |
| Aggregatibacter segnis | 8 |
| Acinetobacter johnsonii | 8 |
| Pseudomonas phage PA16 | 8 |
| Pseudomonas virus phiCTX | 8 |
| Gemella asaccharolytica | 7 |
| Acinetobacter junii | 7 |
| Prevotella denticola | 7 |
| Selenomonas sp oral taxon 126 | 7 |
| Salmonella phage RE 2010 | 7 |
| Porphyromonas endodontalis | 6 |
| Streptococcus pyogenes | 6 |
| Catonella morbi | 6 |
| Capnocytophaga sp oral taxon 332 | 6 |
| Alloprevotella tannerae | 6 |
| Parvimonas sp KA00067 | 6 |
| Treponema medium | 6 |
| Treponema vincentii | 6 |
| Lactococcus lactis | 6 |
| Selenomonas sp oral taxon 138 | 6 |
| Lactobacillus amylovorus | 6 |
| Stenotrophomonas rhizophila | 6 |
| Staphylococcus phage phiN315 | 5 |
| Corynebacterium matruchotii | 5 |
| Moraxella equi | 5 |
| Simonsiella muelleri | 5 |
| Moraxella caprae | 5 |
| Streptococcus gordonii | 5 |
| Pseudomonas sp SHC52 | 5 |
| Stenotrophomonas sp SPM | 5 |
| Stenotrophomonas maltophilia | 5 |
| Pseudomonas geniculata | 5 |
| Pseudomonas phage PAJU2 | 5 |
| Selenomonas sp oral taxon 892 | 4 |
| Pedobacter himalayensis | 4 |
| Acinetobacter nosocomialis | 4 |
| Acinetobacter phage Bphi B1251 | 4 |
| Prevotella copri | 4 |
| Acinetobacter baumannii | 4 |
| Acinetobacter pittii | 4 |
| Acinetobacter sp WC 141 | 4 |
| Neisseria bacilliformis | 4 |
| Fusobacterium necrophorum | 4 |
| Centipeda periodontii | 4 |
| Corynebacterium pseudodiphtheriticum | 4 |
| Serratia marcescens | 4 |
| Streptococcus dysgalactiae group | 4 |
| Enterococcus faecalis | 4 |
| Ottowia sp oral taxon 894 | 4 |
